# Structure, Mechanical, and Lytic Stability of Fibrin and Plasma Coagulum Generated by Staphylocoagulase From *Staphylococcus aureus*

**DOI:** 10.3389/fimmu.2019.02967

**Published:** 2019-12-20

**Authors:** Ádám Z. Farkas, Veronika J. Farkas, László Szabó, András Wacha, Attila Bóta, Lóránt Csehi, Krasimir Kolev, Craig Thelwell

**Affiliations:** ^1^Department of Medical Biochemistry, Semmelweis University, Budapest, Hungary; ^2^Biological Nanochemistry Research Group, Research Centre for Natural Sciences, Budapest, Hungary; ^3^Haemostasis Section, Biotherapeutics Group, National Institute for Standards and Control, Potters Bar, United Kingdom

**Keywords:** coagulase, embolism, endocarditis, fibrin, plasma, *Staphylococcus aureus*

## Abstract

*Staphylococcus aureus* causes localized infections or invasive diseases (abscesses or endocarditis). One of its virulence factors is staphylocoagulase (SCG), which binds prothrombin to form a complex with thrombin-like proteolytic activity and leads to uncontrolled fibrin generation at sites of bacterial inoculation. The aim of this study was to characterize the formation, structure, mechanical properties and lysis of SCG-generated clots. Recombinant SCG was expressed in *Escherichia coli*, purified and the amidolytic activity of its complexes with human prothrombin (SCG-PT) and thrombin (SCG-T) was determined using human thrombin as a reference. Fibrin clots were prepared from purified fibrinogen and human plasma using thrombin, SCG-PT or SCG-T as a coagulase. The kinetics of clot formation and lysis by tissue-type plasminogen activator (tPA) were monitored with turbidimetric assays. Fibrin ultrastructure was examined with scanning electron microscopy and small-angle X-ray scattering (SAXS). Fibrin clot porosity was characterized with fluid permeation assays, whereas the viscoelastic properties and mechanical stability were evaluated with oscillation rheometry. Compared to thrombin, the amidolytic and clotting activity of SCG-PT was 1.6- to 2.5-fold lower on a molar basis. SCG-T had equivalent amidolytic, but reduced clotting activity both on pure fibrinogen (1.6-fold), and in plasma (1.3-fold). The SCG-PT and SCG-T generated fibrin with thicker fibers (10–60% increase in median diameter) than thrombin due to increased number of fibrin protofibrils per fiber cross-section. According to the fluid permeability of the clots SCG-PT and SCG-T promoted the formation of more porous structures. The shear stress resistance in the pure fibrin and plasma clots generated by SCG-PT was significantly lower than in the thrombin clots (243.8 ± 22.0 Pa shear stress was sufficient for disassembly of SCG-PT fibrin vs. 937.3 ± 65.6 Pa in thrombin clots). The tPA-mediated lysis of both pure fibrin and plasma clots produced by SCG-PT or SCG-T was accelerated compared to thrombin, resulting in up to a 2.1-fold increase in tPA potency. Our results indicate that SCG generates a thrombus scaffold with a structure characterized by impaired mechanical stability and increased lytic susceptibility. This proneness to clot disintegration could have implications in the septic embolism from endocardial bacterial vegetation.

## Introduction

*Staphylococcus aureus* is a Gram-positive coccal bacterium, a member of the human normal microbial flora, which can cause a broad palette of pathologies—from localized skin infections to life-threatening invasive diseases (1, 2). The spreading of antibiotic-resistant strains poses an increasing burden on healthcare (3). *S. aureus* infections can be coupled to the formation of abscesses, or valvular vegetation in infective endocarditis (2) which exemplify the interplay between bacterial pathogenic factors and the blood coagulation and innate immunity systems of the host. This pathogen has a remarkable number of virulence factors, some of which are specifically designed to alter and exploit the host coagulation system to its own advantage for propagation in the host organism. One of these factors is staphylocoagulase (SCG), a protein that binds to prothrombin (PT) and the formed SCG-PT complex (staphylothrombin) expresses a thrombin-like proteolytic activity through a non-proteolytic zymogen activation of prothrombin (4, 5).

SCG-PT has a high specificity toward fibrinogen and is unable to bind to other physiological substrates of thrombin, including its plasma inhibitors (6). The resulting uncontrolled fibrin formation is thought to play a role in abscess development and bacterial attachment to cardiac valves in acute endocarditis or vascular implants (6–8). In endocarditis bacteria colonize the endocardium—either following endothelial layer injury or endothelial cell activation—and the bacterial products, as well as the inflammatory process can destroy the heart valve tissue (9). A common complication is the occurrence of septic embolisms (10), but in the absence of data on the characteristics of the fibrin matrix formed by SCG-PT the exact contribution of this fibrin to the fragmentation and embolization of the bacterial vegetations is not clear (11). Our current study addresses the formation, the structural and mechanical properties, as well as the susceptibility to lysis of the SCG-generated fibrin in comparison to the fibrin formed by human thrombin. As SCG interacts not only with PT, but also forms a complex with thrombin (SCG-T), it is of interest to investigate if SCG modifies the characteristics of thrombin-generated fibrin.

## Methods

### Expression of Recombinant SCG

The full-length mature SCG coding region, based on the Newman strain of *S. aureus* [(12); accession code AP009351], was synthesized with an upstream Profinity eXact tag (Biorad, Watford, UK) and cloned into a pET expression vector (performed by VectorBuilder, Shenandoah, TX, USA). Recombinant SCG was expressed as an N-terminal Profinity eXact fusion protein from transformed T7 Express *lys Y Escherichia coli* (New England BioLabs, Hitchin, UK). SCG was purified from inclusion bodies, solubilized with 4 M urea in 100 mM sodium phosphate, pH 7.2, by affinity chromatography using Profinity eXact cartridges (Biorad, Watford, UK) on an AKTA Purifier (GE Healthcare, Amersham, UK). Purified SCG was eluted following cleavage on the column with 25 mM NaF (30 min at room temperature) to remove the Profinity eXact tag. The protein concentration of a pooled batch of SCG was determined to be 380 nM by amino-acid analysis (performed by Alta-Bioscience, Birmingham, UK).

### SCG Activity Assays

Amidolytic activity of human thrombin (NIBSC local reference preparation 01/578, NIBSC, South Mimms, UK), SCG-PT (PT from Calbiochem, La Jolla, CA, USA) and SCG-T was determined in a HEPES buffered solution against the chromogenic substrate for thrombin S-2238 (H-D-Phe-Pip-Arg-pNA; Werfen, Warrington, UK) at 37°C using a plate reader (Molecular Devices, Stamford, CA, USA) to calculate initial hydrolysis rates. Potency estimates for SCG-PT and SCG-T (with SCG at a 1:1.2 molar ratio with PT or T) were calculated relative to thrombin (100 IU/ml) using a parallel line bioassay analysis in Combistats (13). Specific activities were calculated from mean potency estimates and reported as protein concentrations equivalent to 1 IU/ml thrombin activity with the standard error of the mean.

Clotting activity of SCG-PT, SCG-T, and thrombin was determined against 3.0 g/L human fibrinogen (plasminogen depleted, Calbiochem, La Jolla, CA, USA) and human plasma (06/158, lyophilized control plasma; NIBSC, South Mimms, UK) diluted 1:1 in HEPES buffer using a plate reader to monitor absorbance changes at 405 nm. Clotting rates were calculated as the time to reach 50% maximum absorbance from the time courses using a Shiny app for analyzing clotting curves (14). Potency estimates and specific activities were calculated as above.

### Fibrinolysis Assays

Fibrinolysis assays were investigated in microtiter plates on purified fibrin or human plasma clots (100 μl) formed with human thrombin (01/578), SCG-PT and SCG-T (0.5, 5.0, and 50 nM). Each reaction contained purified fibrinogen, or human plasma (06/158) supplemented with purified fibrinogen, to a final concentration of 3.0 g/L, with 60 nM Glu-plasminogen (Hyphen Biomed, Neuville-sur-Oise, France) and a range (5.0–40.0 IU/ml) of tissue-type plasminogen activator (tPA) concentrations (WHO 3rd IS, 98/714, NIBSC, South Mimms, UK). Clotting and lysis were monitored at 405 nm and time courses analyzed using a Shiny app for analyzing clotting and lysis curves (14) to calculate time to 50% lysis from the maximum clotting absorbance. Potency estimates for tPA activity against SCG-(P)T clots were calculated relative to thrombin-formed clots using a parallel line bioassay analysis and semi-weighted combination (13).

### Scanning Electron Microscopy (SEM)

Fibrin was prepared from 2.9 g/L human fibrinogen (plasminogen depleted, Calbiochem, La Jolla, CA, USA) clotted by 0.5/5/50/100 nM of human thrombin, SCG-T or SCG-PT for 4.5 h. All enzymes were diluted in HEPES buffer containing 1 g/L albumin. Recalcified (12.5 mM CaCl_2_) citrated pooled human plasma (Hungarian Blood Supply Service, Budapest, Hungary) was clotted with 5/50/100 nM human thrombin/SCG-T/SCG-PT for 2 h (0.6 g/L fibrinogen in the SCG-T plasma clot; 1.7 g/L fibrinogen in the SCG-PT plasma clot because of different plasma batches). Thereafter clots were fixed according to a previously published method (15). SEM images were taken from 4 to 6 regions of the clots at 20,000× magnification with SEM EVO40 (Carl Zeiss GmbH, Oberkochen, Germany). The diameter of 300 fibrin fibers was measured from each image (altogether 1,200–1,800 measured fiber diameter/clot) using the Image Processing Toolbox of Matlab R2018a (Mathworks, Natick, MA) and the size distribution was evaluated according to a previously described algorithm (15, 16).

### Small Angle X-Ray Scattering (SAXS)

SAXS is a procedure that provides information about the internal structure of the fibrin fibers (17). For these measurements, fibrin clots were prepared in borosilicate capillaries of 1.5 mm outer diameter (0.01 mm wall thickness). After sealing one end of the capillary with flame, 4 μL 250 nM of human thrombin, SCG-T or SCG-PT were applied to its bottom and 16 μL 4.5 mg/mL fibrinogen was added. The content of capillary was stirred for 5 s with a piece of nylon fiber for complete mixing. Finally, the other end of the capillary was sealed with a cylindrical glass plug and quick-setting two-component epoxy resin and SAXS measurements were carried out on “CREDO,” the SAXS camera of the Research Centre of Natural Sciences, Hungary (18). Cu Kα radiation was produced by a GeniX3D Cu ULD integrated beam delivery system equipped with a FOX3D parabolic graded multilayer mirror (Xenocs SA, Sassenage, France). The X-ray beam was shaped using a three-pinhole collimating system (19). SAXS patterns were collected using a Pilatus-300k CMOS hybrid pixel detector (Dectris Ltd, Baden, Switzerland), situated 534 mm downstream from the sample. A second measurement sequence was done using the same procedure, but with 1,305 mm sample-to-detector distance. Raw images were corrected for measurement time, sample self-absorption, geometrical effects and instrumental and environmental background with the on-line data reduction routine implemented in the instrument control software. Intensities have been scaled into absolute units (differential scattering cross-section) using a glassy carbon secondary reference measured along the samples. One-dimensional scattering curves have been derived from the fully corrected and calibrated scattering patterns by azimuthal averaging. The angular dependence of the scattering has been expressed in terms of *q*, the momentum transfer (defined as *q* = 4π sinθ/λ where 2θ is the scattering angle and λ = 0.1542 nm is the X-ray wavelength). After merging the resulting scattering curves from the two sample-to-detector distances, the final datasets covered the range 0.08 < *q* < 6 nm^−1^. Solvent background has been subtracted from all scattering curves.

### Fluid Permeation

To characterize the porosity of the clots fluid permeation studies were performed according to a previously described procedure (20). Fibrinogen or diluted, recalcified plasma (1:1, in HEPES buffer) were clotted in 1 mL pipette tips as described for the SEM measurements above, plasma samples were supplemented with fibrinogen to a 4 g/L final concentration. HEPES buffer was permeated through the clots kept under constant hydrostatic pressure. Porosity (Darcy constant, *K*_*S*_) was determined from the equation (21)

$$\begin{array}{l} K_{S}=\frac{Q^{*}L^{*}\eta }{t^{*}A^{*}P} \end{array}$$

where Q = permeated volume of buffer (cm^3^); η = viscosity of buffer (10^−2^ poise = 10^−7^ N s cm^−2^); L = clot length (1.5 cm); A = average cross-sectional area of the clot (0.09 cm^2^); t = time (s); ΔP = pressure drop (0.170 N cm^−2^ for fibrin clots, 0.054 N cm^−2^ for plasma clots).

### Viscoelasticity and Mechanical Stability Studies

Fibrinogen at 2.9 g/L was mixed with 5/12 nM human thrombin/SCG-T/SCG-PT in a 450 μL final volume and 410 μL of this mixture was quickly transferred into the gap space between the stationary and the oscillating plate of a Haake Rheostress 1 oscillation rheometer (Thermo Scientific, Karlsruhe, Germany). Measurements of the storage modulus (G′) and loss modulus (G″) were performed during a 15 min clotting phase according to a previously published method (22). Following this clotting phase, the flow limit of fibrin gels was determined in the same samples by increasing the applied shear stress (τ) from 0.01 to 2,000.0 Pa stepwise in 300 s, and the viscosity modulus, critical shear stress (τ_0_) were evaluated (22). Recalcified plasma was mixed with 5/10 nM human thrombin/SCG-T/SCG-PT to get a 1.8 g/L final fibrinogen concentration. Before setting the cone to measurement position, mineral oil (white, light, Sigma Aldrich, Budapest, Hungary) was applied around the sample to prevent desiccation of clots. The storage and loss modulus were monitored till G′ reached a plateau, and the flow limit was measured by increasing τ from 0.01 to 500 Pa stepwise in 300 s.

### Statistical Procedures

The optimal continuous theoretical distributions were fitted to the fiber diameter values from the SEM images and these were compared according to Kuiper's test using Monte Carlo simulations as previously described (15, 16). The permeability and viscoelasticity data were analyzed using the non-parametric Kolmogorov-Smirnov test in GraphPad Prism 7®. The level of statistical significance was set at *p* < 0.05.

## Results

### SCG Activity

**Figure 1:**
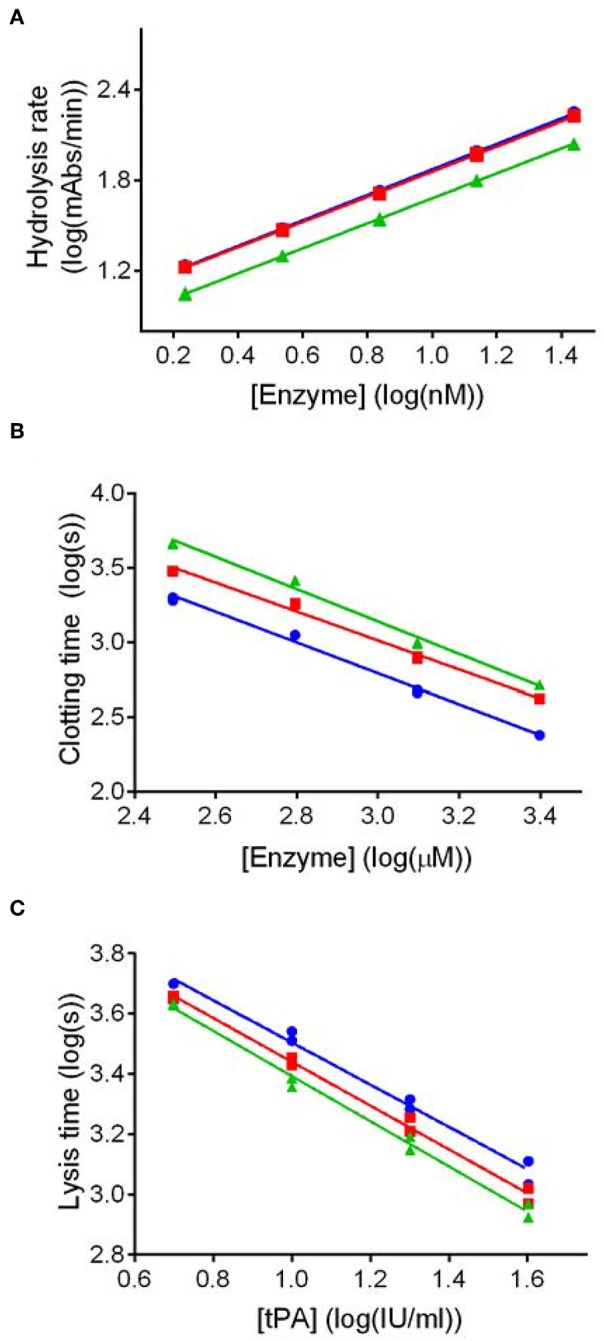
Dose response plots for representative amidolytic, clotting and fibrinolysis assays for thrombin (T), staphylocoagulase-thrombin (SCG-T) and staphylocoagulase-prothrombin (SCG-PT). Potency estimates for SCG-PT (green) and SCG-T (red) were calculated relative to T (blue) using the parallel-line model. The logarithm of doses are represented on the horizontal axes, and the logarithm of responses on the vertical axes. Amidolytic activity **(A)** was calculated using hydrolysis rates (ΔmAbs_405nm_/min) of the chromogenic substrate for thrombin (S-2238) over a range of enzyme concentrations. Clotting activity **(B)** was calculated from turbidimetric clotting times (time to 50% maximum absorbance) over a range of enzyme concentrations. Fibrinolysis activity, represented by tPA potency **(C)**, was calculated using turbidimetric lysis times (from maximum absorbance to 50% lysis) over a range of tPA concentrations in fibrinogen clots formed with 5.0 nM T, SCG-T and SCG-PT. Statistical significance was confirmed using a one-sample *t*-test of log transformed specific activities against the thrombin reference value as a hypothetical mean (*p* ≤ 0.01 in all cases).

**Figure 2:**
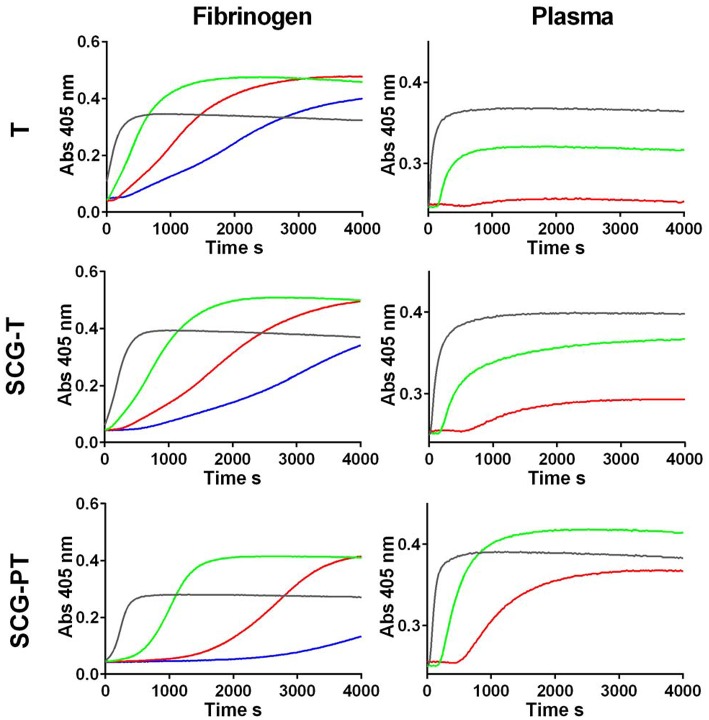
Clotting curves for thrombin (T), staphylocoagulase-thrombin (SCG-T) and staphylocoagulase-prothrombin (SCG-PT) in fibrinogen and plasma. Clot formation was monitored kinetically in microtiter plates at 405 nm over a range of T, SCG-T, and SCG-PT concentrations (0.25 nM, blue; 0.5 nM, red; 1.25 nM, green; 5 nM, black) with purified fibrinogen (3.0 g/L) or human plasma.

Potency estimates for SCG-PT and SCG-T were calculated relative to a local NIBSC reference preparation of human thrombin (01/578; specific activity 9.1 IU/nmol). The parallel line bioassay model was used with a logarithmic transformation of S-2238 hydrolysis rate (ΔmAbs/min) for amidolytic activity, and of time to 50% maximum absorbance for clotting activity, against enzyme concentration (Figures 1A,B). Specific activities (IU/nmol) were calculated for SCG-(P)T using potency estimates and protein concentrations. According to this approach the amidolytic activity of SCG-PT (5.5 IU/nmol [±0.28; *n* = 4]) was ~1.6-fold lower than the reference thrombin (9.1 IU/nmol) and SCG-T (9.2 IU/nmol [±0.6; *n* = 4]). Clotting profiles for T, SCG-T and SCG-PT in both fibrinogen and plasma are shown in Figure 2. SCG-PT clotting activity was ~2.5-fold lower (3.6 IU/nmol [±0.4; *n* = 4]) than thrombin in fibrinogen and 2.2-fold lower (4.2 IU/nmol [±0.3; *n* = 4]) in plasma. SCG-T clotting activity was 1.6-fold lower (5.7 IU/nmol [±0.1; *n* = 4]) than thrombin in fibrinogen and 1.3-fold lower (7.1 IU/nmol [±0.3; *n* = 4]) in plasma.

To conclude, our kinetic data show that the catalytic potency of SCG-PT is significantly lower compared on a molar basis to thrombin resulting in slower clotting of fibrinogen not only in pure systems, but also in the more physiological environment of blood plasma.

### Fibrinolysis

Because the existence of clots *in vivo* depends not only on the rate of their formation, but also on rate of their elimination, we addressed experimentally the susceptibility of SCG-induced clots to lysis. tPA potency estimates of clots formed by SCG-(P)T, with both purified fibrinogen and human plasma, were made relative to thrombin-generated clots using a parallel line analysis of a logarithmic transformation of lysis rates (time to 50% lysis from maximum clot formation) against tPA concentration (Figure 1C). The fibrinolytic potential of clots formed by SCG-(P)T, expressed as % tPA potency relative thrombin-formed clots, is shown in Table 1. In purified fibrinogen, the tPA concentration range used (5.0–40 IU/ml) failed to produce a measurable clot with 0.5 nM SCG-PT, and so a lower range (0.3–2.5 IU/ml) was used. A 2-fold increase in tPA potency was observed at 0.5 nM SCG-PT concentration compared to thrombin. Higher SCG-PT concentrations reduced the fibrinolytic potential to no measurable difference at 50 nM. For SCG-T in fibrinogen, a moderate increase in fibrinolysis was only observed at 5.0 nM, with no apparent difference at 0.5 or 50 nM.

**Table 1:** Fibrinolysis on fibrin and plasma clots.

**Enzyme** ** (nM)**	**Fibrin**		**Plasma**	
	**SCG-T**	**SCG-PT**	**SCG-T**	**SCG-PT**
0.5	108 [99–117]	211 [174–256]	107 [95–121]	145 [132–158]
5	122 [113–132]	142 [132–153]	109 [99–121]	103 [92–114]
50	95 [74–121]	92 [76–111]	158 [144–173]	156 [145–168]

*Fibrinogen or plasma was clotted with the indicated enzyme in the presence of plasminogen and a range of tPA concentrations. Potency estimates for tPA were made for SCG-(P)T clots relative to thrombin-formed clots and are expressed as a percentage of the labeled potency on the WHO 3rd International Standard for tPA (10,000 IU) with 95% confidence limits in brackets (n ≥ 3). Differences are considered statistically different, if the confidence intervals do not overlap*.

In plasma, enhanced fibrinolysis was only observed with SCG-T at 50 nM. For SCG-PT both 0.5 and 50 nM plasma clots showed increased fibrinolysis, with no measurable difference at 5.0 nM compared to thrombin.

As a conclusion, our data show that lysis of both plasma and pure fibrin clots is generally potentiated in SCG-formed clots—with a more accentuated effect with SCG-PT—suggesting a shorter life-span of these clots *in vivo*.

### Clot Structure

Because fibrin structure is an essential determinant of the lytic susceptibility of the clots [reviewed in (23)], our kinetic data on tPA potency prompted the evaluation of the clot structure. To this end we applied SEM, SAXS and pressure-driven permeation techniques.

**Figure 3:**
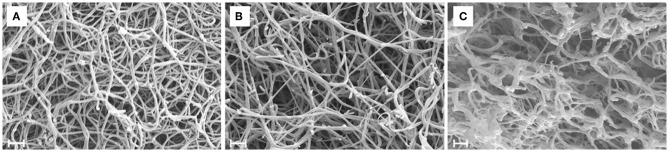
Scanning electron microscopic images of fibrin clots. Fibrin clots were formed with 5 nM human thrombin **(A)**, staphylocoagulase-thrombin **(B)**, and staphylocoagulase-prothrombin **(C)**, and 4–6 images were taken as described in Methods. This was followed by a morphometric analysis, the results of which are presented in Table 2. Scale bar = 1 μm.

According to the SEM images (Figure 3) increasing thrombin concentration resulted in thinner fibrin fibers (Table 2) both in pure fibrin and in plasma clots. These trends were the same when either SCG-T or SCG-PT was used. Generally, the presence of SCG increased the fiber median with both SCG-T and SCG-PT, when compared to thrombin, except for SCG-PT in plasma, which showed fiber thickening only at 100 nM SCG-PT.

**Table 2:** Fiber diameter in fibrin and plasma clots.

**Enzyme (nM)**	**Fibrin**			**Plasma**			
	**Thrombin**	**SCG-T**	**SCG-PT**	Thrombin^**^	**SCG-T**	Thrombin^***^	**SCG-PT**
0.5	121 [92–159]	139* [101–184]	132* [96–182]	–	–	–	–
5	100 [80–127]	114* [89–143]	111* [87–142]	75 [63–90]	106* [87–130]	104 [81–133]	103 [82–128]
50	66 [55–80]	84* [58–106]	86* [69–108]	66 [54–79]	77* [63–93]	92 [73–116]	81* [63–103]
100	51 [42–62]	83* [65–104]	82* [64–104]	66 [54–81]	70* [58–84]	76 [64–90]	79* [64–98]

*Fibrinogen or plasma was clotted with the indicated enzyme and SEM images were taken as illustrated in Figure 3. The diameter of 300 fibrin fibers was manually measured on 4–6 images of each clot. The table presents the median values (in nm) of the measured diameters with bottom and top quartiles in brackets. SCG-T, staphylocoagulase-thrombin; SCG-PT, staphylocoagulase-prothrombin*.

All differences between thrombin and the respective concentration of the SCG-T or SCG-PT are significant (indicated with *) according to Kuiper's test except for the 5 nM thrombin/SCG-PT in plasma.

** *Thrombin control for plasma SCG-T (0.6 g/l fibrinogen)*.

*** *Thrombin control for plasma SCG-PT (1.7 g/l fibrinogen)*.

**Figure 4:**
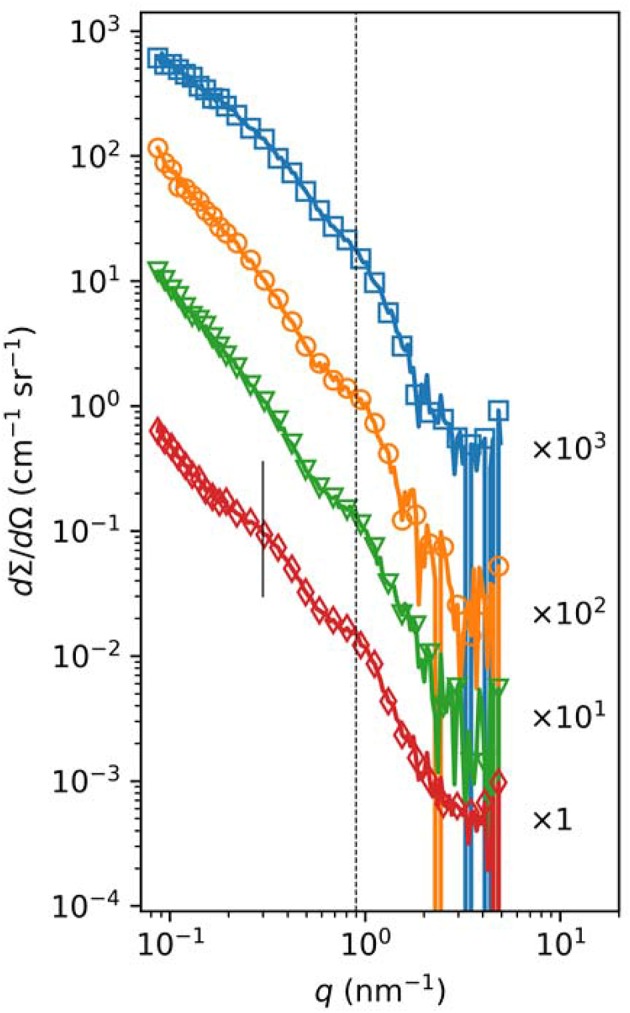
Small-angle X-ray scattering in fibrin clots. Fibrinogen (3.6 mg/mL) was clotted with 50 nM thrombin (orange circles), SCG-T (green triangles), or SCG-PT (red diamonds) under the conditions described in the Methods. Fibrinogen solution was used as a reference (blue squares). The normalized scattering intensity (*d*Σ*/d*Ω) is plotted as a function of the momentum transfer (*q*) and empirical curves are fitted to the raw data, as described in Table S1. Curves are shifted vertically by the indicated factors for better visualization. The dashed vertical line indicates the position of the scattering peak corresponding to periodicity of ~7 nm, whereas the solid vertical line points to the peak for periodicity of about 22 nm observed only in the SCG-PT fibrin.

The increase in fiber diameter could be caused either by larger lateral distance between the protofibrils of polymerizing fibrin monomers or by higher number of monomers per cross-section of the fiber. Our SAXS measurements (Figure 4, Table S1) showed that the scattering peak corresponding to the 7 nm periodicity in the lateral alignment of fibrin monomers (22, 24) was increased in height and area in the SCG-PT-formed clots. The increase in this scattering peak indicated that the average size of the regions containing a consistent 7-nm repeat became typical (larger) suggesting that the typical lateral alignment (and thus density) of protofibrils within the fibers was preserved, but the number of protofibrils per cross-section was increased. Importantly, the structure of SCG-PT-formed fibrin was the only one that showed a SAXS peak for periodicity of about 22 nm (Figure 4) corresponding to higher-order alignment in cluster units of the fibers according to the model of Yang et al. (24).

Although fiber thickness is typically related to clot porosity (25), a more direct characterization of the pore size in the fibrin matrix can be achieved with fluid permeation assays. Using thrombin at increasing concentration, thinner fibers were associated with a smaller pore size (lower *K*_*S*_, Table 3). With SCG-T a reverse trend was noticeable, 100 nM SCG-T resulted in the loosest fibrin matrix. The presence of SCG-T generally increased porosity at least 2-fold except at 0.5 nM. With SCG-PT, the tendencies were more complex; 0.5 and 50 nM concentration of SCG-PT both increased porosity, but at 5 nM SCG-PT strikingly denser clots were found compared to the respective thrombin controls.

**Table 3:** Permeability in fibrin and plasma clots.

**Fibrin clot**	**Thrombin**				**SCG-T**			
	**0.5 nM**	**5 nM**	**50 nM**	**100 nM**	**0.5 nM**	**5 nM**	**50 nM**	**100 nM**
	8.90 ± 3.03	2.57 ± 1.35	2.41 ± 0.88	2.7 ± 1.04	5.18 ± 2.06^*^	6.69 ± 6.00^*^	6.79 ± 4.25^*^	13.40 ± 5.02^*^
	**Thrombin**				**SCG-PT**			
	**0.5 nM**	**5 nM**	**50 nM**		**0.5 nM**	**5 nM**	**50 nM**	
	3.43 ± 2.60	2.24 ± 1.15	0.64 ± 0.29		20.60 ± 1.67^*^	0.50 ± 0.37^*^	5.72 ± 1.97^*^	
**Plasma clot**	**Thrombin**				**SCG-T**			
	**5 nM**	**50 nM**	**75 nM**		**5 nM**	**50 nM**	**75 nM**	
	9.83 ± 4.33	4.57 ± 1.64	4.14 ± 0.69		10.50 ± 4.14	10.20 ± 6.71^*^	8.67 ± 1.89^*^	
	**Thrombin**				**SCG-PT**			
	**5 nM**	**50 nM**	**100 nM**		**5 nM**	**50 nM**	**100 nM**	
	6.91 ± 2.00	4.14 ± 1.36	3.52 ± 0.48		5.91 ± 3.98^*^	5.3 ± 2.91	6.04 ± 1.80^*^	

*Clots were formed using thrombin, staphylocoagulase-thrombin (SCG-T) and staphylocoagulase-prothrombin (SCG-PT) at different concentrations and the permeability constant was determined and compared to the respective thrombin controls. The table presents mean ± SD values of the permeability constant K_S_ (10^-9^ cm^2^) from 3 to 4 independent measurements with 3–5 parallel samples each*.

* *Indicates a p < 0.05 statistical significance compared to the respective thrombin control according to Kolmogorov-Smirnov hypothesis test*.

In plasma clots we detected similar tendencies with SCG-PT (Table 3): 5 nM SCG-PT decreased pore size compared to 5 nM thrombin and a significantly increased porosity was detected at the highest, 100 nM SCG-PT concentration. SCG-T-induced plasma clots also followed the patterns seen in fibrin, but significant differences were observed only at higher concentrations (50 and 75 nM SCG-T).

To summarize our results concerning fibrin structure, we observed a general trend for SCG-PT to produce a fibrin matrix with thicker individual fibers, preserved intrafibrillar density and increased clot porosity.

### Viscoelastic Properties

Because the primary biological function of fibrin is to form a scaffold, the mechanical stability of which is determined by its structure (26), the observed structural features of the SCG-PT-formed fibrin incited the evaluation of its viscoelastic parameters.

**Figure 5:**
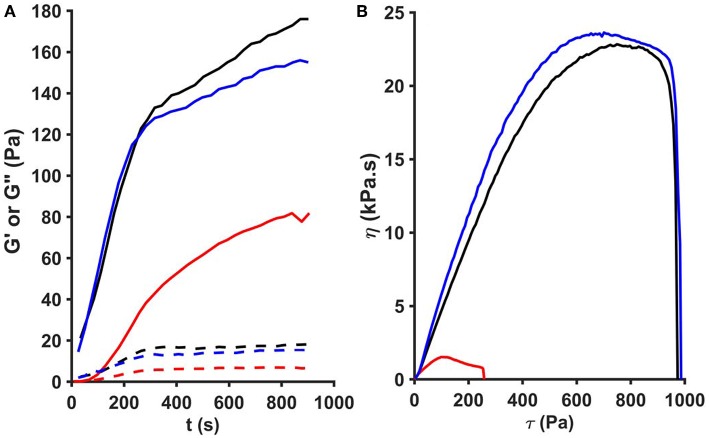
Oscillation rheometer assay of fibrin clots. Fibrinogen (2.9 g/L) was mixed with 5 nM thrombin (black), staphylocoagulase-thrombin (blue), or staphylocoagulase-prothrombin complex (red) and the storage modulus (G′, continuous line) and the loss modulus (G″, dashed line) were measured using an oscillation rheometer **(A)**. Following the 15-min clotting phase, stepwise increasing shear stress (τ) was applied to the clot and viscosity (η) was measured **(B)**. The gel/fluid transition of the clots is characterized by the critical shear stress (τ_0_) at which the value of η falls to zero.

**Figure 6:**
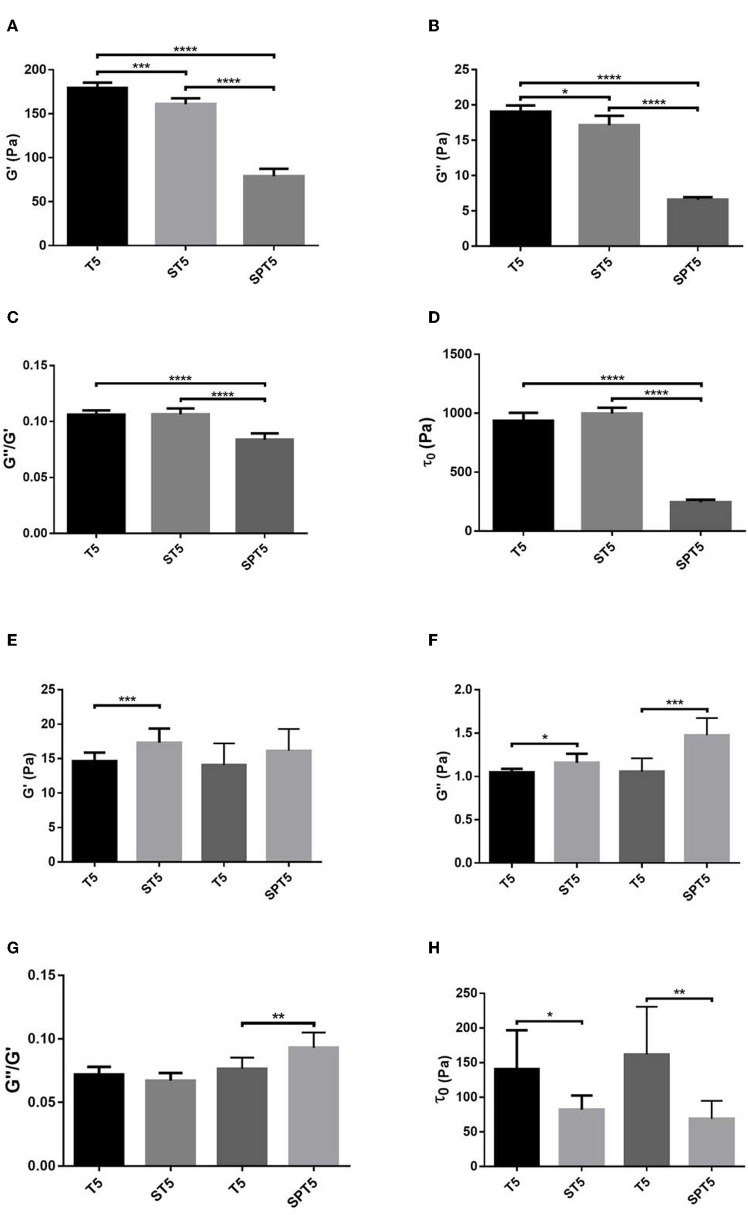
Viscoelastic properties of fibrin and plasma clots. Fibrin **(A–D)** and plasma **(E–H)** coagula were formed as described in Methods and their viscoelastic parameters were determined as illustrated in Figure 5. Bars represent mean and SD following three independent measurements with three replica samples each. G′, Storage modulus; G″, Loss modulus; τ_0_, Critical shear stress; T, thrombin; ST, staphylocoagulase-thrombin; SPT, staphylocoagulase-prothrombin. The numbers after the abbreviations represent the respective concentrations in nM. *p < 0.05; **p < 0.01 ***p < 0.001; ****p < 0.0001 statistical significance according to Kolmogorov-Smirnov hypothesis test.

According to the rheological measurements the presence of SCG (either as SCG-T or SCG-PT) resulted in softening of the fibrin clots (decrease in storage modulus, G′, Figures 5A, 6A). The modifying effect of SCG-PT was stronger generating softer fibrin clots than those formed by thrombin or SCG-T. The loss modulus (G″) of SCG-PT and SCG-T fibrin followed a similar trend of decreased values, representing lower viscosity (Figure 6B). The viscosity component (G″) of the SCG-PT fibrin showed a bigger drop relative to elasticity (G′) (Figure 6C). Similar trends in the modulation of the viscoelastic properties of fibrin were observed at higher enzyme concentrations (Figure S1).

The mechanical stability of fibrin can be characterized with the critical shear stress needed for the disintegration of clots (τ_0_, Figure 5B). The least stable clot was formed by SCG-PT (Figure 6D) with a 4-fold decline in this parameter compared to T or SCG-T. The mechanical stability of T and SCG-T fibrins was rather similar.

Plasma clots showed a different pattern of viscoelastic properties compared to pure fibrin formed by the different clotting enzymes (Figures 6E–H). In contrast to pure fibrin plasma clots generated by SCG-T were consistently more rigid (higher G′, Figure 6E) and SCG-PT also raised significantly the storage modulus at higher enzyme concentration (Figure S2A). The trend of viscosity changes was also the opposite of the one observed for pure fibrin; the loss modulus (G″) was consistently higher for SCG-T and SCG-PT at all evaluated concentrations (Figure 6F, Figure S2B). Concerning the matrix resistance to shear forces (reflected in the values of τ_0_, Figure 6H), both SCG-PT and SCG-T plasma clots were consistently less stable than thrombin clots, mirroring the SCG-PT effect in pure fibrin clots.

## Discussion

The pathogenicity of *S. aureus* is largely dependent on its ability to exploit the host hemostatic system for bacterial colonization through the vasculature (11). Our current study provides novel data on the structural and functional characteristics of the coagulum generated by an isolated key protein (staphylocoagulase) from the *S. aureus* arsenal of factors used to manipulate the hemostatic mechanisms for more efficient pathogenic invasion.

In pure fibrin clots both SCG-PT and SCG-T lead to an altered fibrin meshwork compared to thrombin, suggesting that SCG not only forms staphylothrombin, but also alters the thrombin catalyzed fibrinogen cleavage and/or fibrin polymerization, consistent with the different clotting profiles observed for thrombin and SCG-T. As SCG increased fibrin fiber thickness in a similar manner with both thrombin and PT, it is possible that SCG itself interferes with fibrin polymerization after binding to fibrinogen (8, 27). The equivalence in amidolytic activity between thrombin and SCG-T further implicates SCG-binding to fibrinogen as being responsible for differences in fibrin polymerization and structural properties. The lower amidolytic activity of SCG-PT, and further reduced clotting activity relative to SCG-T suggests the contribution of differences in enzyme activity between thrombin and SCG-PT is additive to the effect of fibrinogen-binding.

Our results showed that when pure fibrinogen was clotted with thrombin, a thicker fiber matrix was associated with a more porous clot structure, which is similar to previously reported data (25). This trend, however, was not seen in fibrin generated by SCG-(P)T. The different trend of SCG-(P)T clot permeability and fiber size could be attributed to an earlier finding that SCG produces a greater amount of background debris compared to thrombin (28), which can explain the formation of thinner fibers—at higher SCG concentrations—without any reduction of the pore size or even allowing for increased porosity. Fibrin clots formed by SCG were more permeable compared to thrombin which probably allows lytic enzymes to penetrate the bacterial coagulum easier (in line with the reported increased tPA potency in SCG clots). At the concentrations used in the rheology studies, both SCG-PT and SCG-T promoted the formation of thicker fibrin fibers (increased median fiber diameter by 10–15 nm). Ryan et al. described the relation between fiber thickness and elasticity (26), and found that there is an optimal, intermediate (75–90 nm) diameter that is also accompanied by an intermediate branchpoint density and results in maximal G′ values. In our study, fiber thickness exceeds this point, thus the expected reduced branching can explain the loss of elasticity of the fibrin structure when SCG was present. However, the most striking difference was the decreased resistance of these clots against shear stress, thus a tendency to disrupt easier and form emboli.

As there are many factors (e.g., plasma proteins, clotting factors) in blood plasma interfering with both fibrinogen and thrombin and altering the structural and mechanical properties of a forming clot, it was of interest to evaluate the coagulum properties in this more complex and physiological system. Our findings suggest that coagulation will be finely tuned in the presence of SCG-T and/or SCG-PT at the site of infection, or in a bacterial vegetation. When SCG-T is dominant, a thicker and more porous matrix is formed compared to thrombin. The structural trends seen in the presence of SCG-PT were more complex, and its effects were concentration dependent. Critical shear stress values were dominantly lower in the presence of SCG than with thrombin, indicating that clots formed by either SCG-T or SCG-PT are easier to disrupt. Interestingly, in plasma SCG increased both elasticity and viscosity of thrombi (when compared to thrombin control), which is the contrary of SCG effects in pure fibrin. In the case of SCG-T, the discrepancy can be explained by the additional interactions of thrombin in plasma [e.g., activation of coagulation factor XIII and the cross-linking of fibers leads to increased elasticity (29)]. SCG-PT, on the other hand, does not interact with other partners of thrombin, thus the changes observed in the viscoelastic parameters might imply a direct effect on the fibrin network, independent of fiber thickness. We also cannot rule out the possibility of other plasma proteins being bound and trapped into the polymerizing matrix in the presence of SCG. Independently from the increased viscoelastic parameters the plasma clots also appeared more fragile (as evidenced by the significantly lower τ_0_ values) than the respective thrombin controls—suggesting an increased risk for thrombus fragmentation and septic embolization—which mirrored our results from the purified fibrin system.

Although the presence of staphylocoagulase in endocardial vegetations was proved by other groups (8), some studies imply that it is not crucial for the development of these structures (30, 31). Nevertheless, our results show that when present, it will have a definite effect on the final properties of the lesion. Our study has its limitations as staphylocoagulase is not a sole player at the site of infection. Lipoteicholic acid (LTA) from *S. aureus* might also change fibrin structure through amyloidogenesis (32). Furthermore, the innate immune system is activated upon a *S. aureus* infection and vegetation formation (11), and it is already reported with *Streptococcus mutans*, that bacterial endocarditis can be accompanied with neutrophil extracellular trap (NET) formation (33). *S. aureus* also has a capability to induce the release of such traps (34), comprised of DNA and histones, that also modify clot structure and increase its mechanical stability (22). Thus, the structure of the vegetations, their fragmentation and the resulting septic embolisms highly depend on the local concentration of clot components (e.g., SCG-T, SCG-PT, NET or even cellular, bacterial components) in different regions of the lesion.

## Data Availability Statement

The datasets generated for this study are available on request to the corresponding author.

## Author Contributions

ÁF, VF, and LC performed the sample preparation for SEM, the permeability and rheometrical studies, analyzed the data, and wrote the manuscript. LS performed the scanning electron microscopic work. AB and AW performed and analyzed the SAXS measurements. CT designed and performed the expression of SCG, performed the clotting and lysis assays and along with KK designed the study, supervised the analysis, and wrote the manuscript. All authors contributed to manuscript revision, read, and approved the submitted version.

### Conflict of Interest

The authors declare that the research was conducted in the absence of any commercial or financial relationships that could be construed as a potential conflict of interest.
