# Supplementary material for: Structure, Mechanical, and Lytic Stability of Fibrin and Plasma Coagulum Generated by Staphylocoagulase From Staphylococcus aureus
Source: Front Immunol. 2019 Dec 20;10:2967. doi: 10.3389/fimmu.2019.02967 (PMC6933771; doi:10.3389/fimmu.2019.02967)
Supplement: Supplementary file 1 [file Data_Sheet_1.pdf]

Supplementary data

Structure, mechanical and lytic stability of fibrin and plasma coagulum generated by staphylocoagulase from *Staphylococcus aureus*

Á. Z. Farkas[1], V. J. Farkas[1], L. Szabó[1], A. Wacha[2], A. Bóta[2], L. Csehi[1], K. Kolev[1], C. Thelwell[3]

[1]Department of Medical Biochemistry, Semmelweis University, Budapest, Hungary

[2]Biological Nanochemistry Research Group, Research Centre for Natural Sciences, Budapest, Hungary

[3]Haemostasis section, Biotherapeutics group, National Institute for Standards and Control, Potters Bar, UK

Correspondence: Krasimir Kolev, Semmelweis University, Department of Medical Biochemistry, 1094 Budapest, Tűzoltó utca 37-47., Hungary, tel.: +36 1 4591500/60035, fax: +36 1 2670031, e-mail: Krasimir.Kolev@eok.sote.hu

In this file we are providing additional information for a better understanding and more detailed view of the results reported in the main text of this article. Figure 1S and 2S represent viscoelastic properties of fibrin and plasma clots formed with a higher concentration (10-12 nM) of human thrombin, staphylocagulase-thrombin (SCG-T) or staphylocoagulase-prothrombin (SCG-PT) complex.

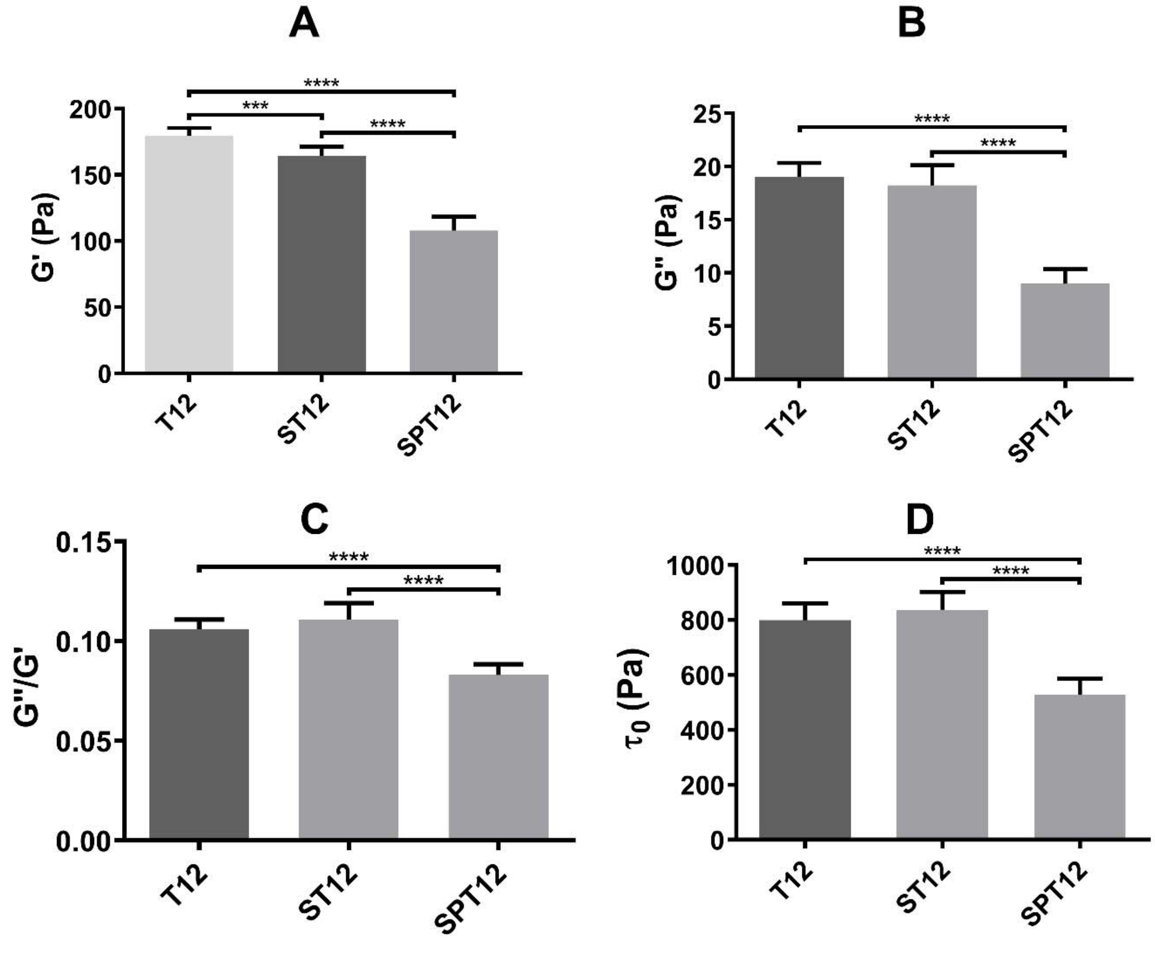

Figure S1. Viscoelastic properties of fibrin clots formed with 12 nM thrombin/staphylocoagulase-trombin or staphylocoagulase-prothrombin complex. The bars represent mean and SD following three independent measurements with three parallel samples. G'=Storage modulus; G"=Loss modulus; τ_0=Critical shear stress; T=thrombin; ST=staphylocoagulase-thrombin; SPT=staphylocoagulase-prothrombin. The numbers after the abbreviations represent the respective concentrations in nM. *** $p<0.001$; **** $p<0.0001$ statistical significance according to Kolmogorov-Smirnov hypothesis test.

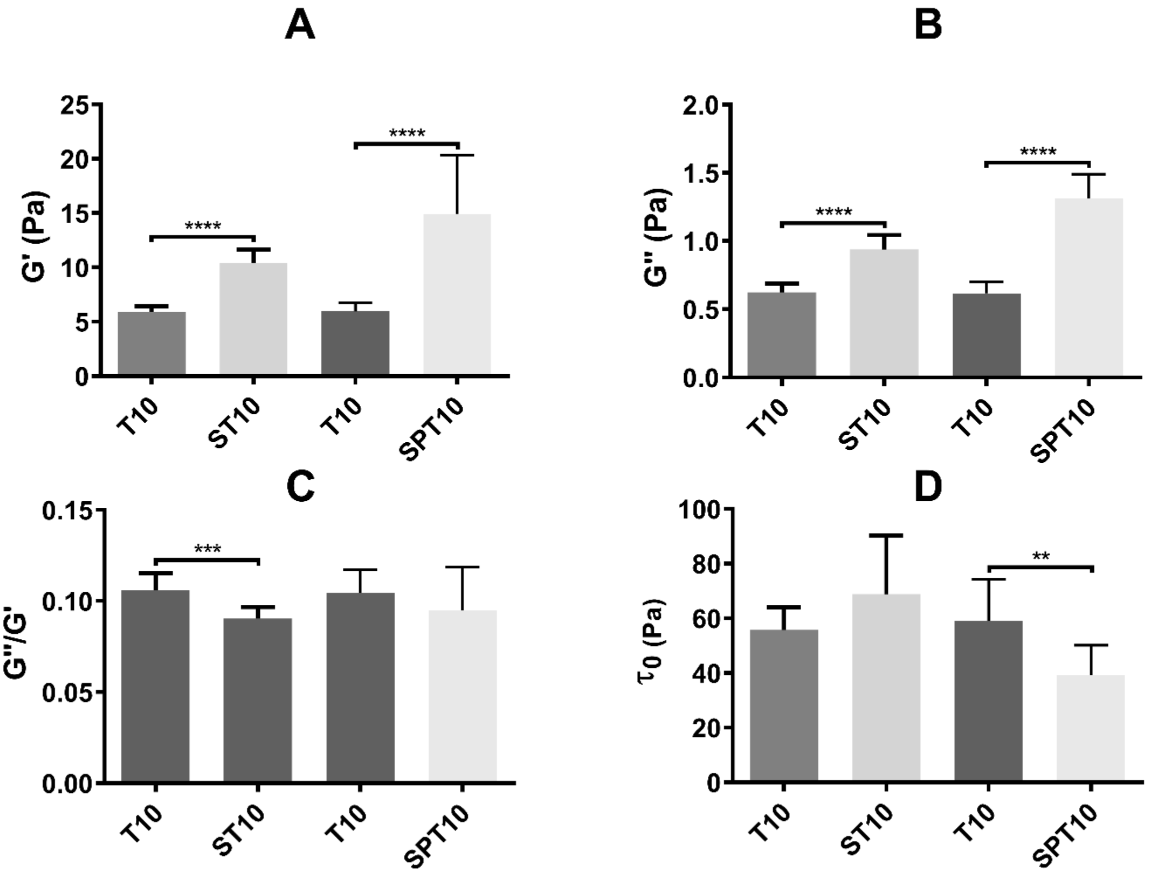

Figure S2. Viscoelastic properties of plasma clots with 10 nM thrombin/staphylocoagulase-trombin or staphylocoagulase-prothrombin complex. The bars represent mean and SD following three independent measurements with three parallel samples. G'=Storage modulus; G"=Loss modulus; τ_0=Critical shear stress; T=thrombin; ST=staphylocoagulase-thrombin; SPT=staphylocoagulase-thrombin. The numbers after the abbreviations represent the respective concentrations in nM. ** $p<0.01$; *** $p<0.001$; **** $p<0.0001$ statistical significance according to Kolmogorov-Smirnov hypothesis test.

Table S1: Parameters of the fitted curves used to interpret the SAXS data of fibrin generated by thrombin, SCG-PT and SCG-T. Quantitative analysis of the SAXS curves shown in Figure 4 was attempted by using an empirical model. The contribution of the fractal behavior of the clots at the beginning of the scattering curve was modelled by a power-law Aq^{α}. Above this baseline, two more Lorentzian peak functions are included, accounting for the two broad peaks: the first one below 0.2 nm^{-1} corresponding to the periodicity of the fibrin cluster units, while the second one around 0.8-0.9 nm^{-1} can be attributed to the average monomer-monomer lateral distance. As seen from the results summarized in the Table, only the position of the second peaks could be obtained with satisfactory certainty. The other quantities – partly because of the shape of the scattering curve, partly due to the correlations between the model parameters – could only be determined with uncertainties much larger than the fit values themselves.

Contribution	Parameter	Unit	Fibrinogen		Thrombin		SCG-T		SCG-PT	
			Value	Uncertainty	Value	Uncertainty	Value	Uncertainty	Value	Uncertainty
**Background**	C	cm^{-1} sr^{-1}	0	(fixed)	0	(fixed)	0	(fixed)	0	(fixed)
	A	cm^{-1} sr^{-1} nm^{-4}	2.73e-05	5.48e-05	5.04e-05	8.30e-05	5.56e-05	1.02e-04	3.75e-05	3.32e-05
	alpha (α)	-	-4	(fixed)	-4	(fixed)	-4	(fixed)	-4	(fixed)
**Peak #1**	Height	cm^{-1} sr^{-1}	0.298	0.365	0.308	0.514	0.363	0.713	0.149	0.114
	Half width	nm^{-1}	0.18	0.07	0.13	0.06	0.12	0.07	0.19	0.12
	Center	nm^{-1}	0.10	0.25	0.11	0.19	0.10	0.19	0.14	0.23
	Area	*cm^{-1} sr^{-1} nm^{-1}*	*0.168*	*0.217*	*0.125*	*0.215*	*0.140*	*0.287*	*0.089*	*0.088*
	Periodicity	*nm*	*62.83*	*154.74*	*58.80*	*103.86*	*62.83*	*120.47*	*44.25*	*71.49*
**Peak #2**	Height	cm^{-1} sr^{-1}	0.004	0.025	0.005	0.020	0.005	0.022	0.006	0.021
	Half width	nm^{-1}	0.13	1.67	0.16	1.20	0.14	0.98	0.16	0.98
	Center	nm^{-1}	0.86	0.88	0.92	0.70	0.88	0.57	0.91	0.54
	Area	*cm^{-1} sr^{-1} nm^{-1}*	*0.002*	*0.022*	*0.002*	*0.020*	*0.002*	*0.019*	*0.003*	*0.021*
	Periodicity	*nm*	*7.28*	*7.47*	*6.86*	*5.23*	*7.16*	*4.62*	*6.88*	*4.11*
